# The eTEP/eTEP-TAR Repair of Ventral Hernias a Study From One Center/ One Surgeon—The First Five Years of Experience

**DOI:** 10.3389/jaws.2024.12796

**Published:** 2024-04-24

**Authors:** Victor G. Radu, Diana Teodora Cucu

**Affiliations:** Life Memorial Hospital—Medlife, Bucharest, Romania

**Keywords:** ventral hernia, laparo-endoscopic retro-muscular repair, eTEP technique, Rives-Stoppa technique, minimally invasive surgery

## Abstract

**Objective:** The objective of this study is analyze the outcomes of retro-muscular repair techniques for ventral hernias performed by a single surgeon in a renowned hernia surgery center.

**Method:** This study involved 197 patients who underwent surgery between May 2016 and December 2021 under the care of a single surgeon (VR). Respecting the indication/contraindications of the eTEP procedure, 197 of 212 patients with ventral hernias underwent eTEP/eTEP-TAR surgery during this period. The cohort consisted of diverse hernia types, including median, lateral, and multiple-site defects. The safety of this approach was evaluated based on postoperative occurrences, where the number of complications accounted for 5% of the cases.

*Results of the study* indicated that there was a significant improvement in the quality of life of patients following the procedure. The assessment, which measured postoperative pain, normal activity, and aesthetics on a 0–10 scale, showed improvement at 2 weeks and 3 months after surgery compared to the preoperative level. However, after a mean of 51.11 months, only one case of recurrence was reported. This recurrence occurred on top of the mesh, 18 months after the initial operation. The follow-up period lasted between 24 and 90 months. Patient monitoring was conducted either in person or over the phone, focusing on quality of life, postoperative pain, and the occurrence of recurrence. In conclusion, the laparo-endoscopic retro-muscular repair of ventral hernias, whether primary or incisional, has shown to yield excellent results in medium and long-term follow-up. The eTEP technique combines the benefits of the Rives-Stoppa technique (considered the gold standard in open ventral hernia repair) with the advantages of minimally invasive surgery.

## Introduction

The Rives-Stoppa procedure is widely recognized as the gold standard for open ventral hernia repair [1]. It involves restoring the linea alba and placing a mesh underneath the rectus muscles. In our center, the cases were resolved using the eTEP-RIVES-STOPPA procedure, which combines the enhanced view provided by laparoscopy (e.g., enhanced view totally extraperitoneal), with the principles of Rives-Stoppa. This approach offers a minimally invasive option with improved outcomes compared to traditional open approaches. In cases involving large hernia defects, lateral hernias, sub-xiphoidal hernias, suprapubic hernias, or complex hernias with multiple defect sites, as well as patients who have undergone previous anterior component separation, a posterior component separation technique may be necessary. This technique involves releasing the transversus abdominis muscle (TAR) alongside other surgical maneuvers to achieve optimal hernia repair and abdominal wall reconstruction [2]. Certainly, in both the eTEP Rives-Stoppa and eTEP-TAR procedures, a polypropylene mesh is placed in the retro-muscular space to provide additional support and augmentation [3].

The goal of the procedure is to restore the linea alba, which is the central tendon of the abdomen, and strengthen the abdominal wall. This is achieved by covering the entire dissected area with a polypropylene mesh [4]. In this study, hernia defects were closed using non-resorbable barbed sutures for restoring the linea alba, and resorbable barbed sutures were used for closing the posterior layer such as the posterior rectus sheaths or peritoneum. Macroporous polypropylene meshes were then placed in the retro-muscular space for augmentation.

## Patients and Method

We reviewed the medical records of patients who underwent laparo-endoscopic retro-muscular repair (specifically, the Rives-Stoppa procedure and abdominal wall reconstruction TAR) for ventral hernias (both primary and incisional). These procedures were performed using a laparo-endoscopic approach (eTEP and eTEP-TAR) between May 2016 and December 2021. The surgeries were conducted by the same surgeon and surgical team at Life Memorial Hospital in Bucharest, Romania. During this period, 212 patients with ventral hernias underwent surgery. However, out of that total, only 197 patients were operated on using the eTEP/eTEP-TAR approach. The remaining 15 patients were not operated on via this access due to contraindications, such as loss of domain (3 cases), poor condition of the overlying skin, infection or pubo-xifoidian scar (8 cases), recurrent hernia after Rives-Stoppa or TAR (4 cases); some cases presented different combination of these conditions.

All the hernias are classified according to EHS criteria [5].

The primary parameter evaluated in terms of postoperative progression has been hernia recurrence. This is routinely assessed during clinical follow-up appointments or through a set of four questions asked during phone follow-up: 1. Do you feel that your hernia is back?, 2. Has any physician told you that your hernia is back?, 3. Do you have a bulge/lump where your hernia used to be?, 4. Do you have any painful area on your abdominal wall? [6]. Other parameters that are measured include the length of hospital stay, occurrence of surgical site issues such as seroma, hematoma, and infection, 30-day readmission following the surgery, and any additional medical or surgical complications that may arise during the follow-up period.

The quality of life was assessed using the VAS. We monitored the level of pain before and after the operation by asking patients about their pain levels while at rest, any restrictions in daily activities (such as walking and climbing stairs), and any cosmetic concerns related to the abdomen and hernia site. Patients provided numerical responses on a scale of 0–10. Chronic pain was defined as pain persisting for more than 3 months after surgery and affecting daily activities. Table 2 and Table 5 respectively depict the pre- and post-operative pain levels.

### Demographic Data of the Patients

This study involved 197 consecutive patients (92 males and 105 females) who were operated on by a single surgeon between May 2016 and December 2021. The mean age of the patients wea 53 years old (median 54 years old).

### Comorbidities of the Patients

Approximately half of the patients were obese (44.7%, n = 88), with a mean BMI of 28.93 and a median BMI of 29.17 (standard deviation 5.5360; range 17.1–45.4). Additionally, 34 patients (17.3%) had diabetes mellitus. When considering cardiovascular and other systemic diseases, the total number of patients with comorbidities was 101 (51.3%, n = 101). Taking in consideration these comorbidities, we calculated the ASA score and obtained the following results: ASA I (a normal healthy patient) 88 patients (44.7%), ASA II (a patient with mild systemic disease) 103 patients (52.3%), ASA III (a patient with severe systemic disease) 6 patients (3%).

### Hernia Characteristics

**FIGURE 1:**
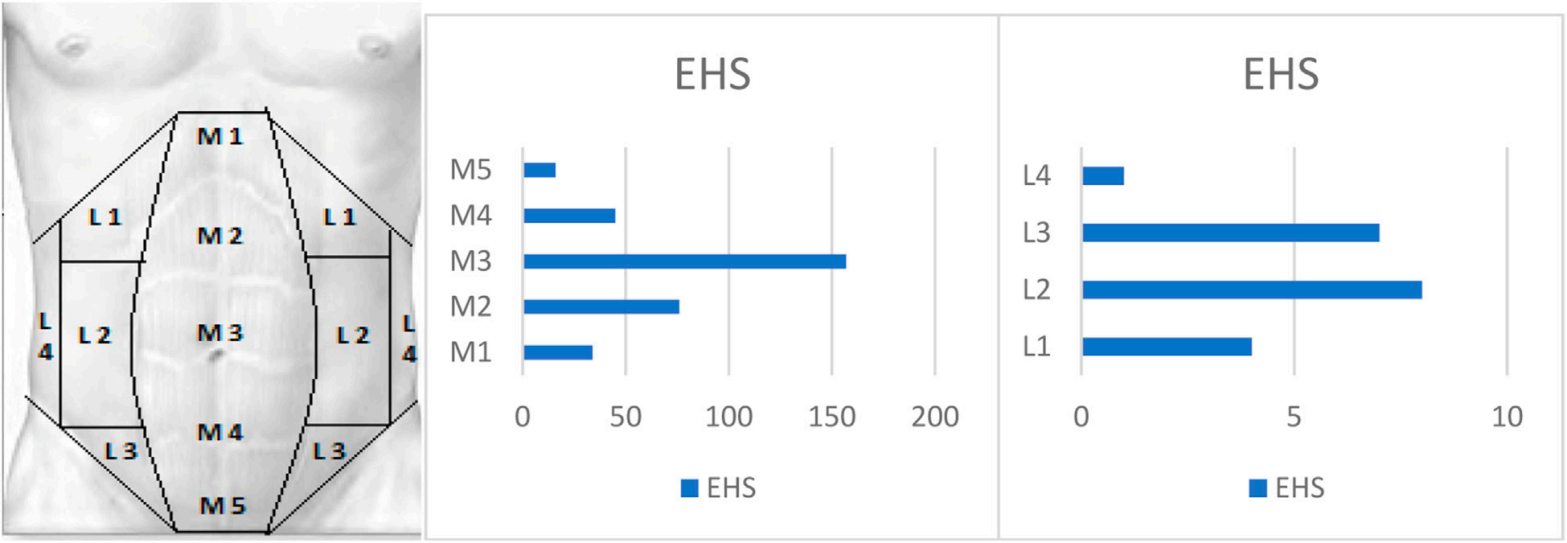
Hernia locations according to the EHS classification are as follows.

The most common type of hernia observed was incisional hernia (62%, n = 122), with the median site of the abdomen being the most frequent location (80.18%) (Figure 1). Out of all the cases, 41 (19.3%) were considered complex incisional hernias, defined as hernias larger than 10 cm in width, with multiple recurrences or multiple site defects [7]. The specific characteristics of the hernias can be found in Table 1.

**TABLE 1:** Hernia characteristics.

	n (%)
Hernia type	
Primary	75 (38%)
Incisional	122 (62%)
Hernia site	
Median	192 (90.6%)
Lateral	5 (2.4%)
Multiple sites	15 (7.1%)
Hernia complexity	
Simple	169 (85.8%)
Complex	28 (14.2%)
Recurrent hernia	
R0	174 (88.3%)
R_n_	23 (11.7%)

The diagnosis of hernias is generally made based on clinical evaluation. In cases of complex incisional hernias or recurrent hernias, imaging diagnostics, such as CT scans, can provide additional information regarding the hernia site, size, abdominal wall structure, and the presence of any previous mesh. In this study, the diagnosis was made using clinical evaluation alone in 140 cases (71.1%). In 55 cases (27.9%), clinical evaluation was supplemented with CT scans, and in 2 cases (1%), ultrasonography was used in addition to clinical evaluation. The length and width of all of the hernias were measured also intraoperatively, as a common step of the procedure,

We assessed the preoperative level of pain during the clinical examination using the 0–10 numerical scale (Visual Analogue Scale—VAS). The majority of patients (74.5%) reported no pain or discomfort (see Table 2).

**TABLE 2:** Preoperative level of the pain using VAS.

VAS	n	Percent
0	149	75.6
1	19	9.6
2	16	8.1
3	7	3.6
4	4	2.0
5	2	1.0
Total	197	100.0

The median defect area is 122 cm^2^, ranging from 6 cm^2^ to 625 cm^2^.

It is well-known that the hernia defect width is the most critical characteristic in closing the defect and reconstructing the abdominal wall, this dimension being one of the characteristics of parietal defects in the EHS classification.

In the patient cohort operated via the eTEP approach, the defect width ranged from 2.5–17 cm in midline incisional hernia, with an average of 6.5 cm, from 7–11 cm in lateral incisional hernia with an average of 7.66 cm, and from 2–7 cm with an average of 3.77 cm in primary ventral hernia (Table 3).

**TABLE 3:** Location and size of the defect; Mesh/defect ratio.

	n	Width (cm) mean (min-max)	Defect area (cm^2^) mean (min-max)	Mesh area (cm^2^) mean (min-max)	Mesh/Defect ratio mean (min-max)
Incisional hernia–midline	105	5.51 (2.5–17)	107 (6–405)	688.25 (195–2025)	10.01 (2.2–66.7)
Ventral hernia–midline	38	3.77 (2–7)	17.74 (4–63)	292.47 (100–600)	28.16 (1.8–99)
Ventral/incisional hernia and diastasis recti - midline	39		82.18 (36–200)	475.9 (200–750)	6.3 (2.8–12.8)
Incisional hernia - lateral	3	7.66 (7–11)	43.25 (18–99)	375 (160–600)	9.06 (6.1–14.2)
Multiple sites defects	12		NA (not applicable)	NA	NA

When it comes to multiple site hernias, these are unique cases that involve at least two distinct hernia sites. In our study, we encountered 12 such cases. The most common scenario for multiple site hernias was a combination of median and lateral (flank) hernias, as well as parastomal hernias with a median component hernia simultaneously. Due to the complexity of these situations, accurately assessing the defect area and determining suitable technical solutions often require the use of two meshes. Therefore, these cases were not included in our analysis table for defect/mesh size.

In 40 cases (20.3%), diastasis recti were found to be associated with ventral hernias. This condition is crucial in determining the size of the mesh to be used. Therefore, regardless of the case, the linea alba is repaired by suturing the anterior sheaths and reinforcing the suture line with an appropriately-sized mesh placed in the retro-rectus space.

In our analysis, the size of the weakness is defined as the actual “diastasis defect” rather than just the hernia defect. For example, if a patient has a small umbilical hernia measuring 2 cm by 2 cm, along with a diastasis recti measuring 5 cm in width and 20 cm in length, we would consider the area of the defect to be 100 cm^2^. In this case, the mesh should be at least 30 cm in length, with the width of the mesh shaped to fit into the retro-rectus space between the two semilunaris lines. In cases involving diastasis, the average length of the defect was 18 cm (minimum 10 cm to maximum 25 cm), and the average length of the mesh used was 28 cm (minimum 10 cm to maximum 30 cm). As for the width of the diastasis, it measured an average of 5 cm (minimum 3 cm to maximum 9 cm), and the mesh was on average 17 cm wide (minimum 10 cm to maximum 25 cm) in order to adequately cover the entire dissected area.

### Procedures

The patient selection process (exclusion criteria) was based on contraindications for the procedure, which included LOD, poor skin condition, recurrence after previous retro-muscular repair, and mesh infection. Out of the total of 212 patients, only 15 underwent open surgery due to contraindications for the eTEP procedure.

Four patients required conversion to an open approach. These conversions were necessary for various reasons: two cases experienced respiratory difficulties during the procedure, one case encountered dense fibrosis within the retro-rectus space, and another case had bowel adhesions to a previous mesh.

The procedures carried out included eTEP Rives-Stoppa (61.9%), eTEP-TAR (36%), and in the converted cases, Rives-Stoppa (1.5%, n = 3) and open TAR (1 case).

Using these procedures, I was able to achieve the main goal of restoring the linea alba, which is the central tendon of the abdomen. For all of the cases, the defect was closed using non-resorbable barbed sutures.

The abdominal wall was reinforced by placing a macroporous, monofilament, medium-weight polypropylene prosthesis in the retro-muscular space. The size of the mesh used in all cases followed the guidelines, with an overlap of the defect of more than 5 cm in each direction (Table 3).

Mesh fixation was common during the early stages of the eTEP procedure. In a total of cases, the mesh was fixed using glue, tackers, or other methods in 25.9% (n = 51) of cases (Table 4).

**TABLE 4:** Mesh fixation.

Mesh fixation	n	%
No fixation	146	74.1
Glue	44	22.3
Resorbable tack	2	1.0
Suture	1	0.5
Mixt (Glue + Tack/Suture)	4	2.0
TOTAL	197	100.0

**FIGURE 2:**
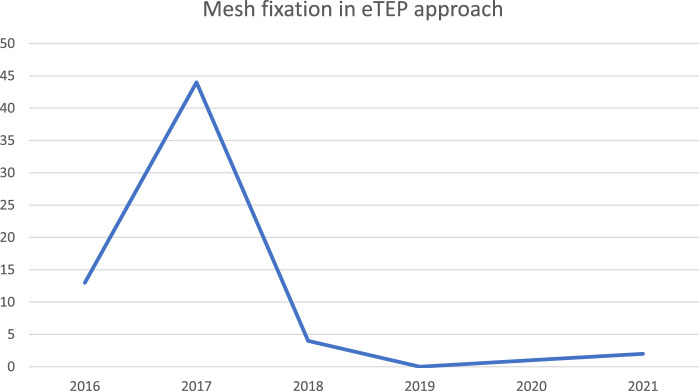
Mesh fixation in eTEP approach.

I want to highlight that, during the initial 2 years of my experience, I used to fix the mesh. However, my current belief is that overlapping the defect is a safe and effective approach for preventing recurrence. Please refer to Figure 2 for a visualization of the mesh fixation in the eTEP approach.

## Results

Throughout my surgeries, I experienced a total of three intraoperative safety events, which accounts for 1.5% of cases. In two instances, I unexpectedly discovered inguinal hernias that were not diagnosed prior to the operation due to the lack of clinical evidence. Additionally, there was one case where a small bowel injury occurred. However, I was able to successfully address and repair all of these issues during the same procedure.

The duration of the operating room (OR) time varied based on the complexity of the hernias (whether they were primary or incisional) and the specific procedure, such as with or without TAR. On average, the OR time was approximately 3 hours (176.37 min).

In the case of primary ventral hernia repair using the eTEP Rives-Stoppa procedure, the average operating room time was 2 hours (121.80 min), while it was 210 min for incisional hernia repair. The mean OR time in eTEP-TAR procedure was 250.54 min (median 240.00 min).

The majority of patients were discharged on the day following their surgery. In order to measure the length of hospitalization (LOS), I recorded the number of hours from the end of the surgery to the time of discharge. The median LOS in this study was found to be 20 h.

The shorter hospital stay can be attributed to the patients experiencing a low level of pain. On average, patients reported taking a median of 2.0 doses (min 0 - max 19) of regular painkillers such as ibuprofen or paracetamol, and no morphine-like medication was required.

Postoperatively, there were a few instances of complications observed. These included 1 case of small bowel obstruction caused by an intraparietal hernia through a ruptured sutured posterior rectus sheath (after eTEP Rives-Stoppa), 1 case of ischemia of the umbilical skin (after eTEP Rives-Stoppa), and 5 cases of retro-muscular hematoma (after eTEP-TAR), with 3 of them requiring re-operation. Additionally, there were 2 cases of suture rupture, one after eTEP Rives-Stoppa and the other after eTEP-TAR, both of which were re-operated on the following day. The postoperative course of the remaining 187 cases (94.9%) was without complications.

I have clinically monitored all of the patients or followed up with them via phone calls for a average period of 51 months (median 52.50 months). The key parameters we have monitored include recurrence (with one patient experiencing recurrence 18 months after surgery, above the mesh) and pain.

To measure the level of discomfort or pain, we utilized the visual analog scale (VAS). The results have been very positive, particularly during the first 3 months after the operation (Table 5).

**TABLE 5:** Postoperative pain VAS score related.

VAS (level of pain)	Frequency	Percent
0	183	92.9
1	6	3.0
2	4	2.0
3	3	1.5
Total	196	99.5
Missing System	1	.5
Total	197	100.0

## Discussion and Conclusion

Results of the study indicated that there was a significant improvement in the quality of life of patients following the MIS retro-muscular procedure. The assessment, which measured postoperative pain, normal activity, and aesthetics on a 0–10 scale, showed improvement at 2 weeks and 3 months after surgery compared to the preoperative level. These results build upon the initial findings published by the same author after the first year of experience, on a cohort of 60 patients. The pain level has consistently been low from the start, with less than 3 doses of analgesics needed per day [8].

However, after a mean of 51.11 months, only one case of recurrence was reported. This recurrence occurred on top of the mesh, 18 months after the initial operation.

Regarding the surgical technique, retro-muscular repair continues to be the preferred method for open ventral hernia repair and is also deemed as the most effective approach in laparoscopic procedures. This technique is associated with fewer surgical site occurrences (SSO) and recurrences, as supported by existing literature [8]. Additionally, patients experience less pain and faster recovery time.

Placing the mesh in the retro-muscular space ensures excellent results in terms of mesh integration with the scar tissue and effective abdominal wall reinforcement. The ideal characteristics of the mesh include polypropylene monofilament, macroporous structure, and medium weight.

Although the eTEP Rives-Stoppa and eTEP-TAR techniques are advanced and require expertise, they combine the principles of the gold standard technique in open ventral hernia repair (Rives-Stoppa) with the benefits of minimally invasive surgery. Consequently, these techniques may yield the best outcomes for hernia treatment.

## Data Availability

The datasets presented in this article are not readily available because the restrictions are related to GDPR: we did not use any personal data of the patients that could lead to the identification of patients (e.g., name, photos, etc.). Requests to access the datasets should be directed to VR, victoradu@gmail.com.
